# Rapid systematic review to identify key barriers to access, linkage, and use of local authority administrative data for population health research, practice, and policy in the United Kingdom

**DOI:** 10.1186/s12889-022-13187-9

**Published:** 2022-06-28

**Authors:** Sowmiya Moorthie, Shabina Hayat, Yi Zhang, Katherine Parkin, Veronica Philips, Amber Bale, Robbie Duschinsky, Tamsin Ford, Anna Moore

**Affiliations:** 1 Cambridge Public Health, Interdisciplinary Research Centre, Forvie Site, Cambridge Biomedical Campus, Cambridge, UK; 2 PHG Foundation, 2 Worts Causeway, University of Cambridge, Cambridge, UK; 3 Department of Public Health and Primary Care, University of Cambridge, Cambridge, UK; 4 University of Cambridge Medical Library, Cambridge, UK; 5 Department of Psychology, University of Northumbria, Newcastle upon Tyne, UK; 6 Department of Psychiatry, University of Cambridge, Herschel Smith Building, Robinson Way, Cambridge, UK; 7 Anna Freud National Centre for Children and Families, London, UK; 8 Cambridgeshire and Peterborough NHS Foundation Trust, Cambridge, Peterborough, UK

**Keywords:** Linked data, Integrated care, Health informatics, Public health

## Abstract

**Background:**

Improving data access, sharing, and linkage across local authorities and other agencies can contribute to improvements in population health. Whilst progress is being made to achieve linkage and integration of health and social care data, issues still exist in creating such a system. As part of wider work to create the Cambridge Child Health Informatics and Linked Data (Cam-CHILD) database, we wanted to examine barriers to the access, linkage, and use of local authority data.

**Methods:**

A systematic literature search was conducted of scientific databases and the grey literature. Any publications reporting original research related to barriers or enablers of data linkage of or with local authority data in the United Kingdom were included. Barriers relating to the following issues were extracted from each paper: funding, fragmentation, legal and ethical frameworks, cultural issues, geographical boundaries, technical capability, capacity, data quality, security, and patient and public trust.

**Results:**

Twenty eight articles were identified for inclusion in this review. Issues relating to technical capacity and data quality were cited most often. This was followed by those relating to legal and ethical frameworks. Issue relating to public and patient trust were cited the least, however, there is considerable overlap between this topic and issues relating to legal and ethical frameworks.

**Conclusions:**

This rapid review is the first step to an in-depth exploration of the barriers to data access, linkage and use; a better understanding of which can aid in creating and implementing effective solutions. These barriers are not novel although they pose specific challenges in the context of local authority data.

**Supplementary Information:**

The online version contains supplementary material available at 10.1186/s12889-022-13187-9.

## Background

Data analysis for improving healthcare is established practice in the United Kingdom (UK). However, the era of big data has led to a greater emphasis on harnessing data from a variety of sources for improved healthcare [1, 2]. This means that increasingly efforts are being made in the UK to access, link and use healthcare data from different sources, including general practice, hospitals and community health services. The value of this approach is especially pertinent to addressing complex health issues such as mental health, which are impacted by a wide variety of factors and where service provision may span several agencies (e.g. health and social care) and may be outside of traditional healthcare settings (e.g. third sector, schools or the workplace). Thus, addressing and improving services for those experiencing complex health problems can be better informed by analysis of data from a range of local public services such as education and social care. In the UK, these data usually sit within local authorities (LA) which are local government organisations responsible for public services in particular areas.

Improving data access, linkage and integration across LAs and other agencies can contribute to improvements in population health. Analysis of health datasets that are routinely collected in the course of public service delivery are an important resource for population health and epidemiological research. They can enable research and analysis to better understand social and biological determinants of health, as well as mechanisms to intervene, either through service or policy development [3, 4].

National policy prioritises appropriate access to, and use of administrative health and LA data [5–7], beyond population health management, to patients, service providers, academics, industry, and policy makers [8]. Such access would optimise health and care outcomes [9], management of integrated pathways [10], cost-effectiveness [11], and service user experience and satisfaction [12, 13]. Furthermore, achieving this is particularly important as inequality has widened over the past 10 years in the UK, and accelerated during 2020 and the COVID-19 pandemic [14, 15]. For health inequalities to be addressed, the Marmot report has recommended the creation of linked administrative databases of health, social care, education, and environmental data that are locally embedded, whole-population, patient-level, granular, and geographically-bound [15].

Finally, the Health and Social Care Bill currently being passed in England will create Integrated Care System Partnerships (ICSP). The Bill includes a requirement for all ICSPs to develop cross-system intelligence functions to support operational and strategic decision-making, underpinned by linked data and accessible analytical resources [16]. Thus, building longitudinal, whole-population, patient-level databases linking social, environment, and health information is a priority to better understand, monitor and enable interventions.

The Cambridge Child Health Informatics and Linked Data (Cam-CHILD) database aims to do this for children’s and young people’s health, by building an anonymized, linked database of health, education, social care and genetic data for the population of Cambridgeshire and Peterborough. This database will be utilised to develop informatics-driven approaches to early identification and intervention for mental health problems in children and young people within the region. Our preliminary analyses of the data requirements for model building indicate a prominent requirement for information relating to social and environmental domains. Many of the required variables are located in existing local authority datasets. However, there are significant barriers to access, linkage and use of this data, with few examples of their routine use to support public health decision making, research, or direct patient care [17].

Understanding the barriers faced in accessing and linking LA data is an important step towards developing solutions to its more efficient use. Thus, the primary aim of this rapid review was to examine reported barriers to the access, linkage and use of such data. A greater understanding of these barriers is important as we embark on the process of bringing together data from a variety of sources. It will also enable others involved in such initiatives, to develop effective and locally driven solutions for use of local authority data.

## Methods

### Search strategy

This review was conducted using systematic review methods and in accordance with the PRISMA statement where possible [18]. The protocol was registered on PROSPERO (www.crd.york.ac.uk/PROSPERO, CRD42021245528) in April 2021.

A systematic literature search was conducted of the following databases; Medline (via Ovid), Embase (via Ovid), Cochrane Library, Global Health (via EBSCO host), PsycINFO (via EBSCO host), CINAHL (via EBSCO host), and Informit Health Collection in January 2021. Searches were date limited from January 2010 to January 2021, using the search terms listed in Table 1 and tailoring them for each database (detailed search terms in Additional file 1). In addition, the PROSPERO registry was searched for newly registered protocols. All results were limited to the UK regions and English language papers.

**Table 1:** Key search terms

Domain	Terms
Data	Social Care” OR “Local Authorit*” OR “Local Government” OR “Public Health” OR “Population Health”).ti,ab,kw. or public health/ or local government/ or population health/
Linkage	“Data linkage” OR “Data sharing” OR “health data” OR “data access” OR “data integration” OR “social care data” OR “medical record linkage” or “integrated care record” or “administrative data”).ti,ab,kw. or medical record linkage/
Barriers and challenges	“Barriers” OR “Challenges” OR “Solutions” OR “Opportunities” OR “Health Inequalities” OR “Problems” OR “Facilitators” or “healthcare disparities” or “health status disparities”).ti,ab,kw. OR healthcare disparities/ or health status disparities/
United Kingdom focus	England” OR “Scotland” OR “Northern Ireland” OR “Wales” OR “Welsh” OR “Scottish” OR “United Kingdom” OR “English” OR “Britain” OR “British” OR “UK”).ti,ab,kw. or exp. United Kingdom**/**

**Fig. 1:**
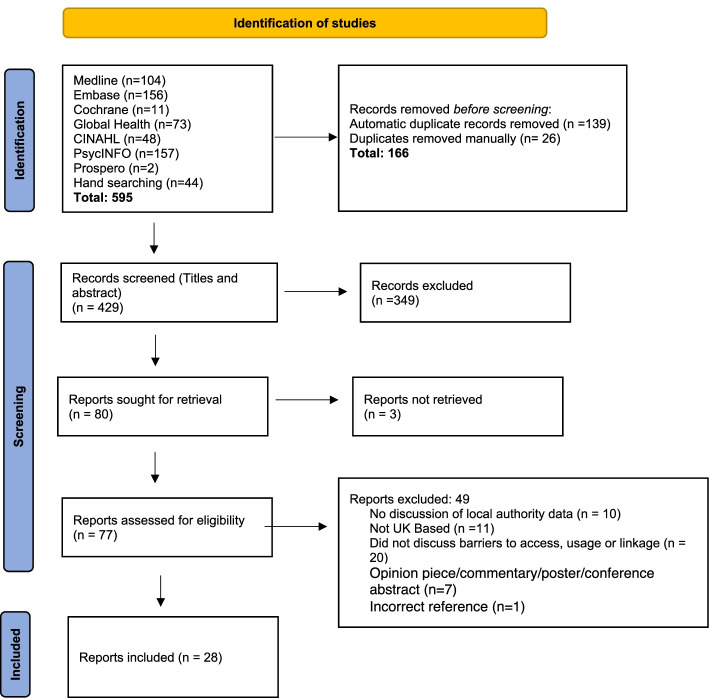
PRISMA flow diagram

The initial database search revealed 551 records. In addition, 44 papers were identified through other sources (hand-searching references, expert communications and grey literature searches). The grey literature search was conducted using the advanced search function in Google and the search terms listed in Table 1. The search was customised by restricting to English language pages, in the UK region and in PDF format. Date restrictions were set as for the initial database searches. Potentially relevant records were identified by examining the first five pages of the search results. Following de-duplication, the titles and abstracts of these records were screened by two authors (S.M. and A.M.) separately. Discussion was used to resolve discrepancies and a final list of 81 articles were identified as eligible for full text screening. Fig. 1 shows the search and selection outcomes for each stage of the review process.

Given the large proportion of policy papers and grey literature documents, papers were not assessed using traditional quality assurance measures.

### Inclusion and data extraction

Five of the authors (S.M., S.H., Y.Z., A.B. and K.P.) assessed full texts of papers for inclusion in the review. Of these papers, 10% (*n* = 5) were assessed by all reviewers to ensure a consistent approach to inclusion. Any publications reporting original research related to barriers or enablers of data linkage of or with local authority data in the United Kingdom were included. Research could be either qualitative or quantitative, and from any phase or study design, including grey literature. Publications focused on countries outside the UK, opinion pieces, letters, commentaries and editorials were excluded. Reviewers met regularly to discuss and resolve any uncertainties or disagreements by consensus.

A standardised form was developed for data extraction. Barriers relating to the following issues were extracted from each paper: funding, fragmentation, legal and ethical frameworks, cultural issues, geographical boundaries, technical, capacity, data quality, security, and patient and public trust. These issues were chosen based on our initial scoping work involving preliminary examination of the literature, which indicated these to be the main areas of discussion. Through a more thorough review, our intention was to elicit a more nuanced understanding of how barriers relating to each of these issues were contributing impacting access, linkage and use of LA data. Data on additional issues beyond these was also extracted, if available. For the most part, the issues cited in papers were the same as those identified in our preliminary scoping work. However, we noted that there was considerable inter-relationship and overlap between these issues. Thus, we subsequently collapsed identified barriers into themes to enable better depiction of the main findings. These themes are: technical capability and data quality; legal and ethical frameworks; funding and capacity; cultural factors; data fragmentation plus public and patient trust.

### Synthesis

A narrative synthesis approach was taken to data analysis. The extracted data was tabulated under the headings indicated above. The analysis included the extent to which barriers to access, linkage and use of local authority data have been examined, the types of publication reporting on this (e.g. grey literature or peer-reviewed), and the main barriers to access, linkage and use of LA data.

## Results

Forty-nine studies were excluded following assessment of full texts. A summary of these articles and reasons for exclusion can be found in Additional file 3. The lack of information on barriers to access, use or linkage of data was the most frequent reason for exclusion. A total of twenty-eight reports were included in the final review, and Table 2 provides a summary of the characteristics of these reports. Of these, 10 were grey literature and the remainder were academic publications. Most (16 publications) of these covered the entire UK, while 6 focused on Scotland, 1 on Wales, and 5 on England (including 2 on London specifically).

**Table 2:** Included studies and characteristics

Number	Author, year	Title	Type	Details	Geography
1	Administrative Data Taskforce (2012) [3]	The UK Administrative Data Research Network: Improving Access for Research and Policy	Grey literature	Independent report offering recommendations to the government following examination of best procedures and mechanisms to make administrative data available for research.	England, Wales, Scotland & Northern Ireland
2	Aitken, M et al. (2016) [19]	Public responses to the sharing and linkage of health data for research purposes: A systematic review and thematic synthesis of qualitative studies	Peer-reviewed	Systematic review of qualitative studies examining public attitudes towards linking and sharing data for research.	Included studies primarily from the UK and North America
3	Atherton, IM et al. (2015) [20]	Barriers and Solutions to Linking and Using Health and Social Care Data in Scotland	Peer-reviewed	Reports the outcome of a meeting of Scottish stakeholders. Covers Scottish experience in linking health and social care data.	Scotland
4	Auditor General for Wales (2018) [21]	The maturity of local government in use of data Archwilydd Cyffredinol Cymru	Grey literature	Report presented to the National Assembly. It assesses whether the local government can capitalise on data that it holds.	Wales
5	Centre for Data Ethics and Innovation (2020) [22]	Addressing trust in public sector data use	Grey literature	Report exploring barriers to data sharing, focusing mainly on citizen trust.	UK
6	Copeland, E (2015) [23]	Small Pieces Loosely Joined: How smarter use of technology and data can deliver real reform of local government	Grey literature	Report describes how local authorities can achieve more by harnessing the principles of digital government and smarter use of technology and data.	UK
7	Davies, JM et al. (2016) [24]	Using routine data to improve palliative and end of life care	Peer-reviewed	Disseminates findings of four workshops on using routinely collected health and social care data in palliative and end of life care.	Covers USA, UK and Europe
8	Downs, JM et al. (2019) [25]	An approach to linking education, social care and electronic health records for children and young people in South London: A linkage study of child and adolescent mental health service data	Peer-reviewed	Describes a study creating a linked dataset of mental health, social and educational records for research in London.	UK, 4 boroughs in South London
9	Comptroller and Auditor General (2019) [26]	Challenges in using data across government	Grey literature	Report by the National Audit Office setting out what the government needs to do with its data to improve services. Reports on challenges and barriers in using data.	UK
10	Office for National Statistics & Government Analysis Function (2020) [5]	Joined up data in government - the future of data linking methods	Grey literature	Cross-government review on data linking methods and making recommendations for government data linkage.	UK
11	Heitmueller, A. et al. (2014) [27]	Developing public policy to advance the use of big data in health care	Peer-reviewed	Article exploring central questions that policy makers should consider when using ‘big data’ in healthcare.	UK
12	Higgins, C & Matthews, K (2020) [28]	Electronic linkage and interrogation of administrative health, social care, and criminal justice datasets: feasibility concerning process and content	Peer-reviewed	Describes a study testing the feasibility of a novel model of electronic linkage and interrogation of large, sensitive, administrative datasets derived from health, social care and criminal justice.	Scotland
13	Office for Statistics Regulation (2018) [29]	Joining Up Data for Better Statistics	Grey literature	Report providing insight into current data linkage activity across government and future opportunities.	UK
14	Iveson, MH & Deary, IJ (2019) [30]	Navigating the landscape of non-health administrative data in Scotland: A researcher’s narrative	Peer-reviewed	Provides a researcher’s narrative of the steps required to gain the various approvals necessary to access and link non-health administrative data for research in social and cognitive epidemiology.	Scotland
15	Kemm, JR et al. (2010) [31]	Social care data in England: What they tell us and what they do not tell us	Peer-reviewed	Reviews the nationally published statistics on adult social services in England, considering their strengths and weaknesses, and how they are produced.	England
16	King, G. et al. (2012) [32]	Boundaries and e-health implementation in health and social care	Peer-reviewed	Describes a study exploring the ways in which structural, professional and geographical boundaries have affected e-health implementation in health and social care.	Scotland
17	Kneale, D. et al. (2016) [33]	Identifying and appraising promising sources of UK clinical, health and social care data for use by NICE	Grey literature	Report prepared by the EPPI-Centre for NICE. Aimed to aid NICE in identifying opportunities for greater use of real-world data within its work.	UK
18	Malomo, F. & Sena, V (2017) [34]	Data Intelligence for Local Government? Assessing the Benefits and Barriers to Use of Big Data in the Public Sector	Peer-reviewed	Article aimed at identifying the main barriers that stop UK local governments from fully benefiting from Big Data.	UK
19	Mansfield, KL. et al. (2020) [17]	Five models for child and adolescent data linkage in the UK: a review of existing and proposed methods	Peer-reviewed	Describes current and hypothesised models for data linkage in relation to key challenges that such work presents.	UK
20	Mourby, MJ. et al. (2019) [35]	Health Data Linkage for UK Public Interest Research: Key Obstacles and Solutions	Peer-reviewed	Outlines key issues which can prevent access to health data for public interest research using three case studies. Also present recommendations.	UK
21	Oyeyemi, A. & Scott, P. (2018) [36]	Interoperability in health and social care: Organisational issues are the biggest challenge	Peer-reviewed	Reports on an investigation of stakeholder views about the major interoperability challenges in health and social care data in England.	England
22	Sexton, A. et al. (2017) [37]	A balance of trust in the use of government administrative data	Peer-reviewed	Describes research examining stakeholder perspectives in relation to administrative data sharing and reuse. Specifically explores the issue of trust in collection, analysis and linkage of data.	UK
23	Local government association (2019) [38]	Local government social care data and interoperability standards: executive summary for social care professionals	Grey literature	Describes findings of a discovery project which aimed to work with councils, the social care system and interoperability suppliers to understand barriers and solutions to support information sharing across social care.	UK
24	Stewart, CH. et al. (2017) [39]	The Scottish school leavers cohort: linkage of education data to routinely collected records for mortality, hospital discharge and offspring birth characteristics	Peer-reviewed	Describes the data sources used to create the Scottish school leavers cohort and highlights the potential of this linked data resource.	Scotland
25	Symons, T. (2016) [40]	Datavores of Local Government: Using data to make services more personalised, effective and efficient	Grey literature	Discussion paper on how data can help councils provide more personalised, effective and efficient services. Part of the Local Datavores Research programme.	UK
26	Wistow, G. et al. (2016) [41]	Why Implementing Integrated Care is so much harder than designing it: experience in North West London. England	Peer-reviewed	Brief paper reporting on initial evaluation of an integrated care programme in North West London.	London
27	Witham, MD. et al. (2015) [42]	Construction of a linked health and social care database resource—Lessons on process, content and culture	Peer-reviewed	Article providing an overview of a successful data linkage process and discussion of potential barriers to executing such projects.	Scotland
28	Muirhead, A. et al. (2016) [43]	The Digital House of Care: information solutions for integrated care	Peer-reviewed	Article describing the development of a digital tool that can be used for integrated health and social care delivery.	Derbyshire

**Fig. 2:**
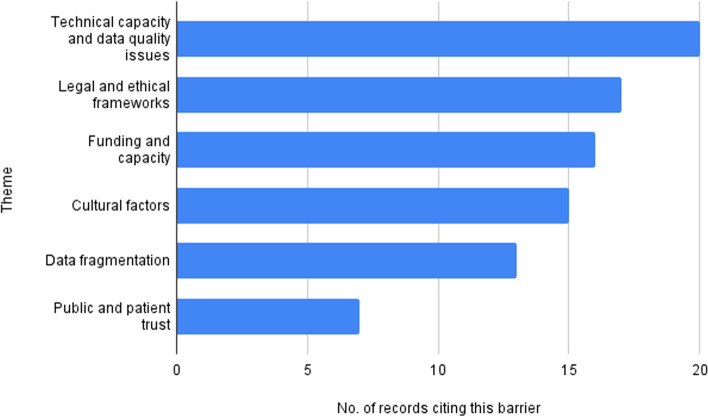
Frequency or reported barriers across citations

Below we provide a synthesis of our main findings for each grouped theme and Fig. 2 provides a summary of the data that was available relating to each (Additional file 2 has tabulated data). Issues relating to technical capability and data quality were cited most often. This was followed by those relating to legal and ethical frameworks. Issue relating to public and patient trust were cited the least, however, there is considerable overlap between this topic and issues relating to legal and ethical frameworks.

### Technical capability and data quality issues

Twenty-one papers cited technical constraints in data linkage. These constraints were either due to legacy systems hindering data sharing across organisations, or the absence of secure methods of data transfer, and issues in creating standardised interoperable systems between organisations. The lack of funding and capacity, as discussed further below also influence the ability to create safe, secure and interoperable systems.

Many reports acknowledged the variable quality of data collected by different organisations, [5, 20–24, 26, 28, 31, 36] with the consequences that effective linkage is much harder to achieve [38, 39], especially as it is a challenge to understand how data are coded and there is potential for missing or unavailable data [20, 37]. Much of local authority data, for example social care data, contain a high proportion of data recorded in an unstructured format [38]. This serves as an additional challenge to its use, with reports that up to 90% of unstructured data is never analysed [21]. Standardisation does not address the problem of how to access and use unstructured data.

A few papers explicitly discussed issues relating to bias and inequalities as acting as a barrier to linkage and use of local authority data. These included overrepresentation [34], as well as underrepresentation of particular groups, for example, women, children, the very elderly, ethnic minorities and those with multiple co-morbidities [33]. In addition, reports also discussed the potential for explicit consent processes to lead to selection bias [3, 19], given differences in which service users are likely to consent to broader use of data [19].

### Legal and ethical frameworks

Both legal frameworks such as the General Data Protection Regulation (GDPR), and ethical principles govern and impact on data access, linkage and use. Existing legal frameworks are designed to address ethical concerns on data processing and require activities involving data to be ethically reviewed. Thus, it can often be difficult to disentangle ethical and legal frameworks with relation to data protection, leading to discussions on this topic being interconnected. As such, we grouped legal and ethical frameworks together for the purposes of data analysis.

Nineteen records identified existing ethical and legal frameworks as a barrier, particularly the complexity of the regulatory landscape pertaining to data protection. Notable variation in the processes for information governance and ethical approvals used to manage compliance with regulatory frameworks were reported between regions and organisations, with inconsistencies in both interpretation and operationalisation. Contributing to this, were the different legal provisions applying to various categories of public sector data, often including sensitive personal data, which may be identifiable, pseudonymised or anonymous [3, 21, 22]. Each must be considered differently by the law, and thus by information governance and ethics committees. Furthermore, the purpose of initial data collection often differ by team and organisation, limiting the ways in which it can subsequently be used.

Accessing or sharing of data, even between public agencies is a complex process that requires a clear understanding of legal frameworks that govern data access and use. Extending this to sharing between agencies for alternative uses, such as research, requires significant expertise which is often not available within local authorities [5, 30, 38]. Lack of familiarity with frameworks that must be applied to enable inter-agency sharing and use contributes to a risk-averse approach [22, 23] and the lack of capacity and resources available hinder problem solving [26]. Understandable efforts to ensure privacy, confidentiality and consent often leads to hesitancy or concerns by organisation in sharing data [3, 5, 17, 19, 22], and where processes were in place, the approval processes, together with the capacity demands within the systems to process these make data access and linkage too time-consuming and resource intensive, and many projects fail [28–30] or are prevented from even starting [3, 21].

### Funding and capacity

Seventeen papers discussed issues related to funding either explicitly or indicated that funding posed a barrier. Reports discussed funding for data linkage initiatives, as well as access to research or strategic funding. In particular, within local authorities the need for funding to build capacity was discussed, for example to upskill staff, and build linked data systems and IT infrastructures, as well as to sustain existing systems [21, 30, 33, 38].

Twelve reports identified capacity constraints stemming from a lack of personnel in government departments with expertise in addressing data access and creating infrastructure for data management. This included the lack of personnel with the expertise to link data within local authorities, and with expertise across sectors, for example health and social care [20, 41]. Linkage across sectors can be particularly challenging and time-consuming. It requires time from those with domain-specific knowledge (e.g. social workers), as well as dedicated informatics expertise. Finding sufficient time for frontline staff to contribute to these projects can be challenging, particularly in an already stretched and busy working environment [36, 41]. In addition, the report by the Local Government Association in 2019 [38] noted that building capacity was hindered by funding cuts to local authorities.

From a research perspective, attempts to create linked datasets were hampered by grant deadlines and high costs associated with accessing data as a result of the many different data access agreements and procedures that need to be navigated [28, 35, 42].

### Cultural factors

Sixteen papers discussed cultural factors as a barrier to creating and utilising linked datasets. These include both individual and organisational cultural factors that impact on data access for subsequent use in linkage initiatives. Data are often owned by different organisations, which have different cultures in relation to willingness to share, and attitudes to data linkage and sharing which influence the ease of data access and linkage [21, 23]. Specifically, willingness to share data, risk aversion and concerns about data breaches were cited as problematic issues [3, 21, 22, 28, 35, 38]. Variation in interpretation of ethical and legal frameworks, as well as the degree of concern about inadvertently going against them impacted on negotiating access to data and in its subsequent linkage and use. The lack of trust between the different parties involved in initiatives to build linked datasets also contributes to issues [3, 21, 22, 28, 29, 35, 38].

Relatedly, the lack of a clear vision for use of data, not treating it as an asset and lack of leadership within organisations [5, 21, 26, 43, 44] also serve to contribute to cultural barriers. This can lead to uncertainty as to what is permissible or desirable by data holders for safe and appropriate use of data.

### Data fragmentation

Fourteen papers discussed data fragmentation and data silos as a barrier to linkage and use of data. The variety of data holders and fragmentation across government departments can contribute to delays in access and use of data [26, 28, 29, 38]. As noted above, cultural factors may influence individual departments’ or organisations’ interpretation of what is permissible or where responsibilities need to be fulfilled to access data. These in turn influence the practicalities of data access such as the requirement for different permissions between and within organisations that need to be granted [3]. In addition, organisational silos can lead to a lack of understanding of available data across local authorities [23, 43].

Related to data fragmentation across data holders, are data silos created by the use of different IT infrastructures within local authorities [17, 21, 23, 26, 33, 38]. The predominance of bespoke and legacy IT systems has led to data being recorded in specific ways, often unique to teams, in a wide range of formats with different coding systems that are not compatible with each other [5, 21, 23]. Furthermore, changes in coding practices within teams or to care processes over time require frequent local system reconfigurations, which are time-consuming and costly [24, 38]. This variety in storage formats leads to difficulties with sharing and linkage even between teams within a council, let alone linkage with other external agencies [20].

Data can also be fragmented across geographical boundaries such as local authorities, counties or countries in the UK. Differences in institutional digital maturity across these boundaries is problematic [3, 21, 26, 32, 38]. The report published in 2012 by the Administrative Data Research UK (ADRUK) noted that addressing this in Scotland and Wales had led to significant gains compared to the rest of the UK enabling a county-wide approach to linkage [3]. Linking between health and social care is significantly more challenging given the minimal use of the National Health Service NHS number within social care across much of England [23, 31, 38]. While there has been significant progress in some areas in the use of the NHS number [38], this has predominantly involved adult social care data, where sharing has been of basic core information such as demographics, allocated case worker and information about services accessed by individuals. To address this, there are examples of linkage methods that used alternative methods of matching records, for example based on hashed de-identified personal identifiers such as birth date and postcode [25, 28, 34, 39]. These methods were shown to be effective for a large proportion of records, but require access either to specialist software, or expensive safe havens that provide linkage services. These approaches require orchestrators to navigate the balance between privacy, confidentiality and scalability [5, 17], and all are resource intensive in terms of cost and time.

Furthermore, variable digital maturity can also lead to implementation of different ethical and legal frameworks as a means to accommodate this, which in themselves can act as a barrier to data access by creating different processes for applying for use of data. Finally, geographical differences in IT infrastructure risks introducing significant regional inequality, for example in remote areas with poor connectivity [32].

### Lack of patient and public trust

Ten reports identified lack of patient and public trust as a barrier to access, linkage and use of local authority data. These reports discussed several factors that contribute to erosion of public confidence and trust in the use of data for research. These include heightened public understanding of rights to privacy, concerns around misuse and exploitation of data, access to private information by commercial organisations, and limited control over uses of their data. This combined with negative publicity about some government data programmes such as care.data underpinned concerns around data sharing and linkage [27, 33]. Furthermore, one report [21] cited that there can be a lack of public trust in local authorities to efficiently manage and achieve full potential from their data. This is because individuals are required to provide the same information multiple times, and there is a lack of clarity on the purpose of each point of data collection.

Overall, negative publicity around administrative data, local authorities’ inefficiency in data exploitation, lack of transparency, and not obtaining informed consent may all reduce public trust in those handling the data, hence reducing public support for data access, use and linkage and use, acting as a barrier. This in turn can also indirectly influence the willingness and extent to which local authorities engage in data linkage and sharing initiatives.

## Discussion

Over the past 10 years, there has been a policy push in the UK towards digitisation and the more effective use of data across organisations to improve healthcare. Data held outside of the NHS, within other agencies such as local authorities, can provide important information that can be used to improve health. However, it is apparent from the papers we reviewed that whilst there has been much progress made to achieve the ambition of joint-working, significant barriers still exist in accessing, linking and using data across these sectors. Addressing these barriers is now imperative to aid the move towards creating integrated care systems.

The objective of this review was to gain a better understanding of the key barriers to accessing, linking and using local authority data for population health research, practice and policy using a systematic approach. Examination of the reports included in this review led to the identification of barriers which were grouped into key themes. Consideration of these barriers and the themes together, suggests there are a core set of interlinked, cross-cutting factors which impact on the ability to access, link and use local authority data for population health research, practice and policy. These are *trust* between different stakeholders; *leadership* to make the best use of data and *capacity* to deliver on data-led initiatives. Although these are not novel, nor specific to local authorities, their impact on the identified barriers is particular to the context in which LAs function.

While local authorities may be considered as a single organisation, in reality many different services and/or agencies contribute to this landscape. In addition, whilst data from these agencies can contribute either directly or indirectly in diverse ways to healthcare, they may not be collected for this purpose. Thus, cross-organisational working is a key element in efforts to harness LA data for health. Cross-organisational working requires trust between data suppliers, holders and users. Sexton et al. [37] discuss how securing public trust is dependent on achieving broader trust between those involved in data initiatives. This can be a challenge given the diversity of stakeholders that may be involved in data initiatives. Legislative and ethical processes are in place to prevent misuse, and are one way of gaining trust and transparency on data uses. However, these are complex and often not clearly communicated, understood or operationalised by many [3, 20, 22, 29]. Underpinning this is ensuring transparency and clarity about how the data will be used. Several of the reports we identified highlighted that where data initiatives involve parties from different organisations, time is needed to build trust and to navigate this landscape in a mutually beneficial fashion.

A repeated theme that related to access, linkage and use of the data was the lack of dedicated leadership for data use and incentives for such leadership within local authorities [5, 21, 34, 44]. In the reviewed literature, there were no examples of a designated senior officer for data issues within an LA. This role was identified as important, and should provide leadership responsibility extending beyond technical requirements into advocacy for the use and reuse of data [21], the budgeting required [5], the adoption of rules and standards [5], establishing the changes in business processes required [5, 34, 40], and navigating what should be linked and shared with whom [5]. Specific gaps were identified in relation to access, linkage, and use, as described above. However, across all domains there was a consistent lack of prioritisation within LAs to embed data skills within workforce strategies. This led to reports of lack of confidence by staff and elected members in the quality of the information being made available for decision-making, reporting and scrutiny [21]. Changes in the political landscape also impact much more on councils and local authorities. This is likely to influence creation of a stable and dedicated leadership for data use.

Many of the barriers we identified were also linked to capacity issues in the form of funding, personnel and skills to deliver on data initiatives. Whilst there is a policy push for more integrated working between health and social care, and there is recognition of the rich contributions that data outside of the health system can make to improving population health, investment in achieving this is not optimal. LA budget cuts and lack of prioritisation of informatics projects means they can struggle to retain the skills required, leading to difficulties in maintaining integrity and accuracy of databases [21]. Budget cuts have often forced IT teams to work across councils, or for IT support to be outsourced, which has led to reduced knowledge of the data systems involved in commissioning and delivery of care [38]. Furthermore, non-integrated technology and legacy systems mean that nearly all local authority IT systems are unique. This raises technology costs, creates duplication and redundancy of capacity, and leads to vendor lock-in, preventing councils from taking advantage of economies of scale. Despite shrinking budgets, LAs are estimated to spend ~ 3–6% on IT, almost twice as much as utility and transport sectors [5, 23, 28]. In spite of this proportionally high spend, a lack of long-term planning to identify future data and intelligence needs means councils are failing to invest in the infrastructure and capability required to enable use of their data for translation into cost-saving initiatives.

The lack of long-term planning extends to consideration of potential secondary uses of data. This can increase costs as additional work needs to be undertaken to make data suitable for sharing and linking, and other expertise such as legal advice and additional technology are required [5]. Lack of awareness of the benefits of linkage means funding to enable interoperability between systems is not a priority. When it is prioritised, a lack of stability of budgets means that novel technologies are not always maintained due to funding cuts. Indeed, some providers decline to operate in the sector due to complexity of the systems, pressures to keep costs down and scarcity of long-term contracts [38]. Collaborative working between councils and other partners can identify opportunities for savings, with examples of the costs associated with data sharing being shared between councils [28]. Government-funded infrastructure projects encouraging collaboration between councils and supporting initial costs of enabling data sharing have been successful. However, it is important that councils concurrently ensure the value is recognised locally and ongoing budgets are allocated to ensure project sustainability [32]. Expanding this sharing also depends on the adoption of common information standards. However, currently many programmes are led by health services, and are often based on medical episodes of care for individuals. This does not always translate to the LA, where care is based more around families, carers and individuals, and is rarely medically-driven [38]. Greater initiation by LAs could help shape data use in a way which benefits them more.

While we were able to find a range of articles discussing barriers to access, linkage and use of data, there are limitations to our work. It is likely that there are reports that we missed, especially in the form of grey literature addressing this issue. Due to the nature of the subject, the search terms used were broad, leading to many non-specific hits. In addition, we employed a rapid review methodology; whilst this followed a systematic review approach, it is less rigorous but was a more pragmatic given time constraints.

Given the heterogeneity of the literature reviewed, the depth of discussion on the barriers to access, linkage and use of data varied, with few academic publications discussing issues relating to access or linkage in detail. This might reflect the focus of such publications in describing research, rather than reporting in detail on barriers faced in conducting research involving linked datasets. The grey literature, especially those reports that were aimed at developing recommendations to move towards the use of linked datasets, provided more substance. However, the focus was not always on population health research.

Finally, the Health and Care Bill is currently being passed to establish Integrated Care Partnerships (ICPs) across England, which will take on the commissioning functions of CCGs and be accountable for NHS spend and performance within the system [16]. These ICPs establish the NHS and local government as equal partners, required to jointly facilitate action to address the wider determinants of health, as well as broader economic development, in order to improve health outcomes. Underpinning these will be digital and data transformation plans that must enable a cross-system approach and provide clear accountability for digital and data use to support population health management, resource planning and performance management. The Bill recognises that improvement requires access to whole-system data. Whilst progress is being made to achieve integration of health and social care data, issues still exist in creating such a system and there remain few examples of successful integration of health and care data. This is also reflected in our experience of developing the Cam-CHILD database, and in a recent paper which describes the issues in relation to creation of LAUNCHES QI which aimed to link audit and national datasets in Congenital Heart Services [45]. Thus, this review provides a starting point for moving towards a better and more nuanced understanding of a broad set of issues impacting on better use of local authority data.

## Conclusions

This rapid review is the first step to an in-depth exploration of the barriers to data access, linkage, and use, a better understanding of which can aid in creating and implementing effective solutions. These barriers are not novel and have been identified by others in relation to health data sharing. However, how these manifest in relation to local authority data and the context in which they are experienced are novel, as are the solutions put forward to address them [3, 20, 38, 43]. As we move towards creation of ICPs, it becomes imperative to share learning on approaches to effectively addressing these barriers, so that we can unlock the potential of LA data for health. The potential for making the most of data across systems has been alluded to by many [2, 8], but achieved by few. As local authorities undertake more research activities [46], joint efforts to address these barriers and achieve effective data sharing in a safe and acceptable way is important to enhance research that feeds into service provision and improves population health.

## Supplementary Information

**Additional file 1.** Search terms.

**Additional file 2.** Summary of included studies against each theme.

**Additional file 3.**

## Data Availability

The authors confirm that the data supporting the findings of this review are available within the article or in supplementary materials.
