# Supplementary material for: Rapid systematic review to identify key barriers to access, linkage, and use of local authority administrative data for population health research, practice, and policy in the United Kingdom
Source: BMC Public Health. 2022 Jun 28;22:1263. doi: 10.1186/s12889-022-13187-9 (PMC9241330; doi:10.1186/s12889-022-13187-9)
Supplement: Supplementary file 1 — Additional file 1. Search terms. [file 12889_2022_13187_MOESM1_ESM.docx]

**Additional File 1: Search terms**

**Medline via Ovid**

(“Social Care” OR “Local Authorit*” OR “Local Government” OR “Public Health” OR “Population Health”).ti,ab,kw. or public health/ or local government/ or population health/

AND

(“Barriers” OR “Challenges” OR “Solutions” OR “Opportunities” OR “Health Inequalities” OR “Problems” OR “Facilitators” or “healthcare disparities” or “health status disparities”).ti,ab,kw. OR healthcare disparities/ or health status disparities/

AND

(“England” OR “Scotland” OR “Northern Ireland” OR “Wales” OR “Welsh” OR “Scottish” OR “United Kingdom” OR “English” OR “Britain” OR “British” OR “UK”).ti,ab,kw. or exp United Kingdom/

AND

(“Data linkage” OR “Data sharing” OR “health data” OR “data access” OR “data integration” OR “social care data” OR “medical record linkage” or “integrated care record” or “administrative data”).ti,ab,kw. or medical record linkage/

**Embase via Ovid**

(“Social Care” OR “Local Authorit*” OR “Local Government” OR “Public Health” OR “Population Health”).ti,ab,kw. or social care/ or public health/ or population health/

AND

(“Barriers” OR “Challenges” OR “Solutions” OR “Opportunities” OR “Health Inequalities” OR “Problems” OR “Facilitators” or “healthcare disparities” or “health status disparities”).ti,ab,kw. or health disparity/

AND

(“England” OR “Scotland” OR “Northern Ireland” OR “Wales” OR “Welsh” OR “Scottish” OR “United Kingdom” OR “English” OR “Britain” OR “British” OR “UK”).ti,ab,kw. or exp United Kingdom/

AND

(“Data linkage” OR “Data sharing” OR “health data” OR “data access” OR “data integration” OR “social care data” OR “medical record linkage” or “integrated care record” or “administrative data”).ti,ab,kw. or data integration/ or medical record linkage/

**Cochrane Library**

(“Social Care” OR “Local Authorit*” OR “Local Government” OR “Public Health” OR “Population Health”):ti,ab,kw or MeSH descriptor: [Local Government] this term only or MeSH descriptor: [Public Health] this term only or MeSH descriptor: [Population Health] this term only

AND

(“Barriers” OR “Challenges” OR “Solutions” OR “Opportunities” OR “Health Inequalities” OR “Problems” OR “Facilitators” or “healthcare disparities” or “health status disparities”):ti,ab,kw or MeSH descriptor: [Healthcare Disparities] this term only or MeSH descriptor: [Health Status Disparities] this term only

AND

(“England” OR “Scotland” OR “Northern Ireland” OR “Wales” OR “Welsh” OR “Scottish” OR “United Kingdom” OR “English” OR “Britain” OR “British” OR “UK”):ti,ab,kw or MeSH descriptor: [United Kingdom] explode all trees

AND

(“Data linkage” OR “Data sharing” OR “health data” OR “data access” OR “data integration” OR “social care data” OR “medical record linkage” or “integrated care record” or “administrative data”):ti,ab,kw or MeSH descriptor: [medical record linkage] this term only

**Global Health via EBSCO** **host**

TI (“Social Care” OR “Local Authorit*” OR “Local Government” OR “Public Health” OR “Population Health”) OR AB (“Social Care” OR “Local Authorit*” OR “Local Government” OR “Public Health” OR “Population Health”) OR DE "public health”

AND

TI (“Barriers” OR “Challenges” OR “Solutions” OR “Opportunities” OR “Health Inequalities” OR “Problems” OR “Facilitators” or “healthcare disparities” or “health status disparities”) OR AB (“Barriers” OR “Challenges” OR “Solutions” OR “Opportunities” OR “Health Inequalities” OR “Problems” OR “Facilitators” or “healthcare disparities” or “health status disparities”)

AND

TI (“England” OR “Scotland” OR “Northern Ireland” OR “Wales” OR “Welsh” OR “Scottish” OR “United Kingdom” OR “English” OR “Britain” OR “British” OR “UK”) OR AB (“England” OR “Scotland” OR “Northern Ireland” OR “Wales” OR “Welsh” OR “Scottish” OR “United Kingdom” OR “English” OR “Britain” OR “British” OR “UK”) or DE “UK” or DE "England" OR DE "Wales" OR DE "Scotland" OR DE "Northern Ireland"

AND

TI (“Data linkage” OR “Data sharing” OR “health data” OR “data access” OR “data integration” OR “social care data” OR “medical record linkage” or “integrated care record” or “administrative data”) or AB (“Data linkage” OR “Data sharing” OR “health data” OR “data access” OR “data integration” OR “social care data” OR “medical record linkage” or “integrated care record” or “administrative data”)

**CINAHL via EBSCO host**

TI (“Social Care” OR “Local Authorit*” OR “Local Government” OR “Public Health” OR “Population Health”) OR AB (“Social Care” OR “Local Authorit*” OR “Local Government” OR “Public Health” OR “Population Health”) OR (MH “Public Health”)

AND

TI (“Barriers” OR “Challenges” OR “Solutions” OR “Opportunities” OR “Health Inequalities” OR “Problems” OR “Facilitators” or “healthcare disparities” or “health status disparities”) OR AB (“Barriers” OR “Challenges” OR “Solutions” OR “Opportunities” OR “Health Inequalities” OR “Problems” OR “Facilitators” or “healthcare disparities” or “health status disparities”)

AND

TI (“England” OR “Scotland” OR “Northern Ireland” OR “Wales” OR “Welsh” OR “Scottish” OR “United Kingdom” OR “English” OR “Britain” OR “British” OR “UK”) OR AB (“England” OR “Scotland” OR “Northern Ireland” OR “Wales” OR “Welsh” OR “Scottish” OR “United Kingdom” OR “English” OR “Britain” OR “British” OR “UK”) OR (MH "United Kingdom+")

AND

TI (“Data linkage” OR “Data sharing” OR “health data” OR “data access” OR “data integration” OR “social care data” OR “medical record linkage” or “integrated care record” or “administrative data”) or AB (“Data linkage” OR “Data sharing” OR “health data” OR “data access” OR “data integration” OR “social care data” OR “medical record linkage” or “integrated care record” or “administrative data”)

PsycINFO via EBSCO host

TI (“Social Care” OR “Local Authorit*” OR “Local Government” OR “Public Health” OR “Population Health”) OR AB (“Social Care” OR “Local Authorit*” OR “Local Government” OR “Public Health” OR “Population Health”) or KW (“Social Care” OR “Local Authorit*” OR “Local Government” OR “Public Health” OR “Population Health”) OR (DE "Public Health")

AND

TI (“Barriers” OR “Challenges” OR “Solutions” OR “Opportunities” OR “Health Inequalities” OR “Problems” OR “Facilitators” or “healthcare disparities” or “health status disparities”) OR AB (“Barriers” OR “Challenges” OR “Solutions” OR “Opportunities” OR “Health Inequalities” OR “Problems” OR “Facilitators” or “healthcare disparities” or “health status disparities”) or KW (“Barriers” OR “Challenges” OR “Solutions” OR “Opportunities” OR “Health Inequalities” OR “Problems” OR “Facilitators” or “healthcare disparities” or “health status disparities”) or DE "Health Disparities"

AND

TI (“England” OR “Scotland” OR “Northern Ireland” OR “Wales” OR “Welsh” OR “Scottish” OR “United Kingdom” OR “English” OR “Britain” OR “British” OR “UK”) OR AB (“England” OR “Scotland” OR “Northern Ireland” OR “Wales” OR “Welsh” OR “Scottish” OR “United Kingdom” OR “English” OR “Britain” OR “British” OR “UK”) OR KW (“England” OR “Scotland” OR “Northern Ireland” OR “Wales” OR “Welsh” OR “Scottish” OR “United Kingdom” OR “English” OR “Britain” OR “British” OR “UK”)

AND

TI (“Data linkage” OR “Data sharing” OR “health data” OR “data access” OR “data integration” OR “social care data” OR “medical record linkage” or “integrated care record” or “administrative data”) or AB (“Data linkage” OR “Data sharing” OR “health data” OR “data access” OR “data integration” OR “social care data” OR “medical record linkage” or “integrated care record” or “administrative data”) or KW (“Data linkage” OR “Data sharing” OR “health data” OR “data access” OR “data integration” OR “social care data” OR “medical record linkage” or “integrated care record” or “administrative data”)

**Informit Health Collection**

TI=(“Social Care” OR “Local Authorit*” OR “Local Government” OR “Public Health” OR “Population Health”) OR AB=(“Social Care” OR “Local Authorit*” OR “Local Government” OR “Public Health” OR “Population Health”)

AND

TI=(“Barriers” OR “Challenges” OR “Solutions” OR “Opportunities” OR “Health Inequalities” OR “Problems” OR “Facilitators”) OR AB=(“Barriers” OR “Challenges” OR “Solutions” OR “Opportunities” OR “Health Inequalities” OR “Problems” OR “Facilitators” or “healthcare disparities” or “health status disparities”)

AND

TI=(“England” OR “Scotland” OR “Northern Ireland” OR “Wales” OR “Welsh” OR “Scottish” OR “United Kingdom” OR “English” OR “Britain” OR “British” OR “UK”) OR AB=(“England” OR “Scotland” OR “Northern Ireland” OR “Wales” OR “Welsh” OR “Scottish” OR “United Kingdom” OR “English” OR “Britain” OR “British” OR “UK”)

AND

TI=(“Data linkage” OR “Data sharing” OR “health data” OR “data access” OR “data integration” OR “social care data” OR “medical record linkage” or “integrated care record” or “administrative data”) OR AB=(“Data linkage” OR “Data sharing” OR “health data” OR “data access” OR “data integration” OR “social care data” OR “medical record linkage” or “integrated care record” or “administrative data”)

**PROSPERO**

Two searches: public AND data linkage AND United Kingdom; public AND health record linkage AND United Kingdom

Limited to English language and published in the last 10 years.
