# Supplementary material for: Rapid systematic review to identify key barriers to access, linkage, and use of local authority administrative data for population health research, practice, and policy in the United Kingdom
Source: BMC Public Health. 2022 Jun 28;22:1263. doi: 10.1186/s12889-022-13187-9 (PMC9241330; doi:10.1186/s12889-022-13187-9)
Supplement: Supplementary file 2 — Additional file 2. Summary of included studies against each theme. [file 12889_2022_13187_MOESM2_ESM.docx]

Additional file 2:

**Supplementary Table: Summary of included studies against each theme**

| **Theme** | **No. of records citing this barrier** | **Citations** |
| --- | --- | --- |
| Technical capacity and data quality issues | 21 | Administrative Data Taskforce (2012); Atherton, IM. et al. (2015); Auditor General for Wales (2018); Centre for Data Ethics and Innovation (2020); Copeland, E. (2015); Davies, JM. (2016); Downs, JM. et al. (2019); Comptroller and Auditor General (2019); Office for National Statistics & Government Analysis Function (2020); Higgins, C. & Matthews, K. (2020); Office for Statistics Regulation (2018); Iveson, MH. & Deary, IJ. (2019); Kemm, JR. et al. (2010); Kneale, D. et al (2016); Malomo, F. & Sena, V (2017); Oyeyemi, A. & Scott, P. (2018); Sexton, A. et al. (2017); Local Government Association (2019); Stewart, CH. et al. (2017); Symons, T. (2016); Witham, MD. et al. (2015); Muirhead, A. et al. (2016) |
| Legal and ethical frameworks | 19 | Administrative Data Taskforce (2012); Aitken, M. et al. (2012); Auditor General for Wales (2018); Centre for Data Ethics and Innovation (2020); Copeland, E. (2015); Davies, JM. (2016); Comptroller and Auditor General (2019); Office for National Statistics & Government Analysis Function (2020); Higgins, C. & Matthews, K. (2020); Office for Statistics Regulation (2018); Iveson, MH. & Deary, IJ. (2019); Mansfield, KL. et al (2020); Mourby, MJ. et al. (2019); Malomo, F. & Sena, V (2017); Oyeyemi, A. & Scott, P. (2018); Local Government Association (2019); Symons, T. (2016); Wistow, G. et al. (2016); Witham, MD. et al. (2015) |
| Funding and capacity | 17 | Administrative Data Taskforce (2012); Atherton, IM. et al. (2015); Auditor General for Wales (2018); Centre for Data Ethics and Innovation (2020); Comptroller and Auditor General (2019); Office for National Statistics & Government Analysis Function (2020); Higgins, C. & Matthews, K. (2020); Office for Statistics Regulation (2018); Iveson, MH. & Deary, IJ. (2019); Kneale, D. et al. (2016); Mourby, MJ. et al. (2019); Malomo, F. & Sena, V (2017); Oyeyemi, A. & Scott, P. (2018); Local Government Association (2019); Symons, T. (2016); Wistow, G. et al. (2016); Witham, MD. et al. (2015) |
| Cultural factors | 16 | Administrative Data Taskforce (2012); Atherton, IM. et al. (2015); Auditor General for Wales (2018); Centre for Data Ethics and Innovation (2020); Copeland, E. (2015); Davies, JM. (2016); Comptroller and Auditor General (2019); Office for National Statistics & Government Analysis Function (2020); Higgins, C. & Matthews, K. (2020); Iveson, MH. & Deary, IJ. (2019); Malomo, F. & Sena, V (2017); Mourby, MJ. et al. (2019); Sexton, A. et al. (2017), Local Government Association (2019); Witham, M.D et al. (2015); Muirhead, A. et al. (2016) |
| Data fragmentation | 14 | Administrative Data Taskforce (2012); Auditor General for Wales (2018); Copeland E. (2015); Davies JM. (2016); Comptroller and Auditor General (2019); Higgins, C. & Matthews, K. (2020); Office for Statistics Regulation (2018); King, G. et al. (2020); Kneale, D. et al. (2016); Malomo, F. & Sena, V (2017); Mourby, MJ. et al. (2019); Local Government Association (2019); Wistow, G. et al. (2016); Witham, MD. et al. (2015) |
| Public and patient trust | 10 | Administrative Data Taskforce (2012); Atherton, IM. et al. (2015); Auditor General for Wales (2018); Centre for Data Ethics and Innovation (2020); Copeland, E. (2015); Comptroller and Auditor General (2019); Office for National Statistics & Government Analysis Function (2020); Office for Statistics Regulation (2018); Heitmueller, A. et al. (2014); Kneale, D. et al. (2016) |
