# Supplementary material for: Rapid systematic review to identify key barriers to access, linkage, and use of local authority administrative data for population health research, practice, and policy in the United Kingdom
Source: BMC Public Health. 2022 Jun 28;22:1263. doi: 10.1186/s12889-022-13187-9 (PMC9241330; doi:10.1186/s12889-022-13187-9)
Supplement: Supplementary file 3 — Additional file 3. [file 12889_2022_13187_MOESM3_ESM.docx]

Additional file 3:

**Supplementary Table: Excluded articles and reasons for exclusion**

Full text articles excluded, with reasons (n = 49)

| Reason for exclusion | Number of articles | Citations |
| --- | --- | --- |
| No discussion of local authority data | 10 | Ainsworth & Buchan (2015)(1)  Fleming et al. (2014) (2)  Ford et al. (2019)(3)  Galetsi et al. (2019)(4)  Hodgson et al. (2020)(5)  Hopf et al. (2014a) (6)  Hurt et al. (2019)(7)  Jordan et al. (2019)(8)  Lyons et al. (2016) (9)  Stoye et al. (2020)(10) |
| Not UK-based | 11 | Bernstein et al. (2015)(11)  Barone et al. (2017)(12)  Jutte et al. (2011)(13)  Lee (2020)(14)  Manyika et al. (2011)(15)  McCormick (2018)(16)  Rizi & Roudsari (2013)(17)  Roos et al. (2017)(18)  van Panhuis et al. (2014)(19)  Wellcome Trust (2015)(20)  Williams et al. (2019)(21) |
| Did not discuss barriers to access, use or linkage | 20 | Baldacchino et al. (2010)(22)  Baranyi et al. (2020)(23)  Black et al. (2015)(24)  Cheetham et al. (2019)(25)  Egan et al. (2016)(26)  Fone et al. (2016)(27)  Green et al. (2019)(28)  Hagger-Johnson (2016)(29)  Harron et al. (2017)(30)  Henderson et al. (2021)(31)  Jayatunga et al (2019)(32)  John et al. (2014)(33)  Muirhead et al (2016) (34)  Murtagh et al. (2018)(35)  National Data Guardian for Health and Social Care (2020)/Caldicott (2020)(36)  Scheibner et al. (2020)(37)  Scourfield et al. (2019)(38)  Siew et al. (2016)(39)  Tod et al. (2019)(40)  Williamson et al. (2017)(41) |
| Opinion piece/commentary/poster/conference abstract | 7 | Drake et al. (2019)(42)  Hodgson et al. (2019)(43)  Hopf et al. (2010)(44)  Hopf et al. (2011)(45)  Hopf et al. (2012)(46)  Hopf et al. (2014b) (47)  O'Doherty et al. (2016)(48) |
| Incorrect reference | 1 | John et al (2015)(49) |

Reference list of excluded articles

1. Ainsworth J, Buchan I. Combining Health Data Uses to Ignite Health System Learning. Methods of Information in Medicine. 2015;54(6):479-87.

2. Fleming LE, Haines A, Golding B, Kessel A, Cichowska A, Sabel CE, et al. Data mashups: Potential contribution to decision support on climate change and health. International Journal of Environmental Research and Public Health. 2014;11(2):1725-46.

3. Ford E, Boyd A, Bowles JKF, Havard A, Aldridge RW, Curcin V, et al. Our data, our society, our health: A vision for inclusive and transparent health data science in the United Kingdom and beyond. Learning Health Systems. 2019;3(3):e10191.

4. Galetsi P, Katsaliaki K, Kumar S. Values, challenges and future directions of big data analytics in healthcare: A systematic review. Social Science & Medicine. 2019;241.

5. Hodgson S, Fecht D, Gulliver J, Daby HI, Piel FB, Yip F, et al. Availability, access, analysis and dissemination of small-area data. International Journal of Epidemiology. 2020;49(Suppl. 1):i4-i14.

6. Hopf Y, Bond C, Francis J, Haughney J, Helms P. Important issues with data linkage: A consensus seeking exercise. International Journal of Pharmacy Practice. 2014;22:39-40.

7. Hurt L, Ashfield-Watt P, Townson J, Heslop L, Copeland L, Atkinson MD, et al. Cohort profile: HealthWise Wales. A research register and population health data platform with linkage to National Health Service data sets in Wales. BMJ Open. 2019;9(12).

8. Jordan J-A, McCann M, Katikireddi SV, Higgins K. Harmonising alcohol consumption, sales and related outcomes data across the UK and Ireland: an insurmountable barrier to policy evaluation? Drugs: Education, Prevention & Policy. 2019;26(5):385-93.

9. Lyons RA, Turner S, Lyons J, Walters A, Snooks HA, Greenacre J, et al. All Wales Injury Surveillance System revised: development of a population-based system to evaluate single-level and multilevel interventions. Injury Prevention. 2016;22(Suppl. 1):i50-i5.

10. Stoye G, Zaranko B, Shipley M, McKee M, Brunner EJ. Educational Inequalities in Hospital Use Among Older Adults in England, 2004-2015. Milbank Quarterly. 2020;98(4):1134-70.

11. Bernstein JA, Friedman C, Jacobson P, Rubin JC. Ensuring public health’s future in a national-scale learning health system. American Journal of Preventive Medicine. 2015;48(4):480-7.

12. Barone A, Bernal-Delgado E, Kuchinke W, van Staa T, Cunningham J, Lettieri E, et al. Health Data for Public Health: Towards New Ways of Combining Data Sources to Support Research Efforts in Europe. Yearbook of medical informatics. 2017;26(1):235-40.

13. Jutte DP, Roos LL, Brownell MD. Administrative Record Linkage as a Tool for Public Health Research. Annual Review of Public Health. 2011;32(1):91-108.

14. Lee JW. Big Data Strategies for Government, Society and Policy-Making. JOURNAL OF ASIAN FINANCE ECONOMICS AND BUSINESS. 2020;7(7):475-87.

15. Manyika J, Chui Brown M, B. J B, Dobbs R, Roxburgh C, Hung Byers A. Big data: The next frontier for innovation, competition and productivity. McKinsey Global Institute. 2011(June):156-.

16. McCormick EV. Public health and population health: Leveraging electronic health record data for local population health surveillance: ProQuest Information & Learning; 2018.

17. Rizi SA, Roudsari A. Development of a public health reporting data warehouse: lessons learned. Studies in health technology and informatics. 2013;192:861-5.

18. Roos LL, Walld R, Burchill C, Roos NP, Nickel N. Linkable administrative files: Family information and existing data. LONGITUDINAL AND LIFE COURSE STUDIES. 2017;8(3):262-80.

19. Van Panhuis WG, Paul P, Emerson C, Grefenstette J, Wilder R, Herbst AJ, et al. A systematic review of barriers to data sharing in public health. BioMed Central Ltd.; 2014. p. 1144-.

20. Wellcome T, Green E, Ritchie F, Mytton J, Webber DJ, Deave T, et al. Enabling Data Linkage to Maximise the Value of Public Health Research Data: full report. 2015.

21. Williams F, Oke A, Zachary I. Public health delivery in the information age: the role of informatics and technology. SAGE Publications Ltd; 2019. p. 236-54.

22. Baldacchino A, Crome IB, Zador D, McGarrol S, Taylor A, Hutchison S, et al. Recording of clinical information in a Scotland-wide drug deaths study. Journal of Psychopharmacology. 2010;24(9):1289-98.

23. Baranyi G, Cherrie M, Curtis S, Dibben C, Pearce JR. Neighborhood crime and psychotropic medications: a longitudinal data linkage study of 130,000 scottish adults. American Journal of Preventive Medicine. 2020;58(5):638-47.

24. Black C, Evans J, Lowbrigde P, Rodger J, Gordon S, Hall S, et al. Implementing best practice in data governance to enable health improvement through high quality data linkage research. European Journal of Epidemiology. 2013;28(1):S144-S5.

25. Cheetham M, Redgate S, van der Graaf P, Hunter R. Local Authority Champions of Research Project : A Report for the Health Foundation Study team2019. 1-111 p.

26. Egan M, Kearns A, Katikireddi SV, Curl A, Lawson K, Tannahill C. Proportionate universalism in practice? A quasi-experimental study (GoWell) of a UK neighbourhood renewal programme's impact on health inequalities. Social Science & Medicine. 2016;152:41-9.

27. Fone D, Dunstan F, White J, Webster C, Rodgers S, Lee S, et al. Change in alcohol outlet density and alcohol-related harm to population health (CHALICE). BMC Public Health. 2012;12(1):428-.

28. Green N, Sherrard-Smith E, Tanton C, Sonnenberg P, Mercer CH, White PJ. Assessing local chlamydia screening performance by combining survey and administrative data to account for differences in local population characteristics. Scientific reports. 2019;9(1):7070.

29. Hagger-Johnson G. Opportunities for longitudinal data linkage in Scotland. Scottish Medical Journal. 2016;61(3):136-45.

30. Harron K, Dibben C, Boyd J, Hjern A, Azimaee M, Barreto ML, et al. Challenges in administrative data linkage for research. Big data & society. 2017;4(2):2053951717745678-.

31. Henderson DAG, Atherton I, McCowan C, Mercer SW, Bailey N. Linkage of national health and social care data: a cross-sectional study of multimorbidity and social care use in people aged over 65 years in Scotland. Age and Ageing. 2020.

32. Jayatunga W, Asaria M, Belloni A, George A, Bourne T, Sadique Z. Social gradients in health and social care costs: Analysis of linked electronic health records in Kent, UK. Public Health. 2019;169:188-94.

33. John A, Dennis M, Kosnes L, Gunnell D, Scourfield J, Ford DV, et al. Suicide information database-cymru: A protocol for a population-based, routinely collected data linkage study to explore risks and patterns of healthcare contact prior to suicide to identify opportunities for intervention. BMJ Open. 2014;4 (no pagination).

34. Muirhead A, Ward DG, Howard B. The Digital House of Care: information solutions for integrated care. Journal of Integrated Care. 2016;24(5/6):237-48.

35. Murtagh MJ, Blell MT, Butters OW, Cowley L, Dove ES, Goodman A, et al. Better governance, better access: Practising responsible data sharing in the METADAC governance infrastructure. Human Genomics. 2018;12(1).

36. National Data G. Survey report Information sharing to support direct care. 2020.

37. Scheibner J, Ienca M, Kechagia S, Troncoso-Pastoriza JR, Raisaro JL, Hubaux J-P, et al. Data protection and ethics requirements for multisite research with health data: a comparative examination of legislative governance frameworks and the role of data protection technologies†. Journal of Law and the Biosciences. 2020;7(1).

38. Scourfield J, Corliss C, Wijedasa D, Robling M, Clayton V. Overview of Administrative Data on Children's Social Care in England. 2019.

39. Siew ED, Basu RK, Wunsch H, Shaw AD, Goldstein SL, Ronco C, et al. Optimizing administrative datasets to examine acute kidney injury in the era of big data: Workgroup statement from the 15<sup>th</sup> ADQI Consensus Conference. Canadian Journal of Kidney Health and Disease. 2016;3(1).

40. Tod E, McCartney G, Fischbacher C, Stockton D, Lewsey J, Grant I, et al. What causes the burden of stroke in Scotland? A comparative risk assessment approach linking the Scottish Health Survey to administrative health data. PLoS ONE. 2019;14(7):e0216350-e.

41. Williamson AE, Ellis DA, Wilson P, McQueenie R, McConnachie A. Understanding repeated non-attendance in health services: A pilot analysis of administrative data and full study protocol for a national retrospective cohort. BMJ Open. 2017;7(2).

42. Drake A, Pollitt A, Smith L, Sklar E. Data awareness for sending help (DASH): Policy opportunities & challenges. Emergency Medicine Journal. 2019;36 (1):E12.

43. Hodgson S, Crellin E, Syder K, Sadarangani S, Padmanabhan S. Area-level linkages to enrich primary care electronic health data for research. Pharmacoepidemiology and Drug Safety. 2019;28 (Supplement 2):246-7.

44. Hopf Y, Bond C, Haughneya J, Helms P. Linkage of routinely collected NHS data to create a paediatric pharmacovigilance database: Opinions of national stake holders. International Journal of Pharmacy Practice. 2010;18:77-8.

45. Hopf Y, Bond C, Haughney J, Helms P. Data linkage for pharmacovigilance - Seeking the views and opinions of healthcare professionals. International Journal of Pharmacy Practice. 2011;19:48.

46. Hopf Y, Bond C, Francis J, Haughney J, Helms P. Data linkage for pharmacovigilance: Opinions of healthcare professionals on the secondary use of paediatric administrative NHS data. International Journal of Pharmacy Practice. 2012;20:15-6.

47. Hopf YM, Bond C, Francis J, Haughney J, Helms PJ. Views of healthcare professionals to linkage of routinely collected healthcare data: a systematic literature review. Journal of the American Medical Informatics Association. 2014;21(e1):e6-e10.

48. O'Doherty KC, Christofides E, Yen J, Bentzen HB, Burke W, Hallowell N, et al. If you build it, they will come: Unintended future uses of organised health data collections. BMC Medical Ethics. 2016;17.

49. John A, Dennis M, Kosnes L, Gunnell D, Scourfield J, Ford DV, et al. Suicide information database-Cymru: A protocol for a population-based, routinely collected data linkage study to explore risks and patterns of healthcare contact prior to suicide to identify opportunities for intervention. BMJ Open. 2015;4(11).
